# Unveiling the epigenetic and stress-responsive function of histone H1 in plants

**DOI:** 10.1080/15592324.2026.2677313

**Published:** 2026-05-21

**Authors:** Min Jae Bae, Shah Zareen, Hyeseon Yun, Ahktar Ali, Zein Eddin Bader, Kisuk Park, Nassem Albakri, Young Jun Jung, Dae-Jin Yun, Junghoon Park

**Affiliations:** a School of Advanced Biotechnology, Global Plant Stress Research Center, Konkuk University, Seoul, Republic of Korea; b Institute of Natural Medicine, University of Toyama, Toyama, Japan; c National Institute of Ecology, Maseo-myeon, Seocheon-gun, Republic of Korea

**Keywords:** Histone H1, nucleosome, chromatin architecture, TE, stress adaptation

## Abstract

Environmental stresses can induce epigenetic alterations, particularly in chromatin accessibility and DNA methylation patterns, which orchestrate gene expression in *Arabidopsis thaliana*. Many molecular mechanisms with diverse aspects have been reported to play a role, but a thorough understanding is still required. The linker protein Histone H1 (H1) plays an essential role in modulating the chromatin architecture to control gene expression. H1 stabilizes the nucleosome structure and promotes higher-order chromatin compaction. It interacts with chromatin modifiers to influence nucleosome accessibility, thereby regulating transcriptional events. H1 variants exhibit distinct spatiotemporal expression patterns and play key roles in stress adaptation. Under abiotic stress, the depletion of H1 is associated with dynamic DNA demethylation at transposable elements and altered histone marks. For instance, upon H1 depletion under drought or heat stress, shifts in H3K9me2 deposition have been observed, indicating a dual role for H1 in promoting genome stability and facilitating stress-induced transcriptional reprogramming. This review summarizes the emerging insights into H1 molecular mechanisms, crosstalk between noncoding RNAs, and phase-separated condensates. We also discuss the unresolved questions on H1 variant specificity and evolutionary conservation, as well as their potential applications in improving crop resilience through epigenetic engineering.

## Introduction

In plants, epigenetic regulation plays a pivotal role in stress responses by modulating gene expression without altering the DNA sequence. This layer includes mechanisms such as DNA methylation, histone post-translational modifications (PTMs; e.g., methylation and acetylation), incorporation of histone variants, and chromatin remodeling, which dynamically influence chromatin structure and accessibility.^1-3^

These mechanisms enable rapid, reversible transcriptional shifts between open (permissive) and closed (repressive) states, coordinated by enzymes such as HATs/HDACs that recruit transcriptional machinery, with CpG methylation fine-tuning TF binding for development and resilience.^1^ ^,^ ^4^ Epigenetic marks also confer “stress memory,” priming enhanced responses to recurrent challenges, transiently within a lifespan.^3^ ^,^ ^5^ Plant-specific pathways, including RdDM and active demethylation (e.g., DME-mediated), underpin this inheritance of adaptive traits.^6-13^

A comprehensive understanding of these intricate epigenetic controls offers promising avenues for the advancement of innovative crop improvement strategies. Epigenetically modified organisms represent a promising alternative with enhanced stress tolerance without the incorporation of foreign DNA, potentially avoiding the lesser acceptability commonly associated with conventional genetically modified organisms.^11^ Numerous studies have demonstrated improved stress resilience in crops such as rice, cotton, and tomato achieved through targeted DNA methylation and via small RNA-based pathways, highlighting the translational potential of epigenetic knowledge in agriculture.^14-16^ A comprehensive understanding of stress-signaling networks in plants coupled with epigenetic regulation is critical for advancing sustainable cropping practices in the face of increasing environmental challenges.^1^

**Figure 1.**
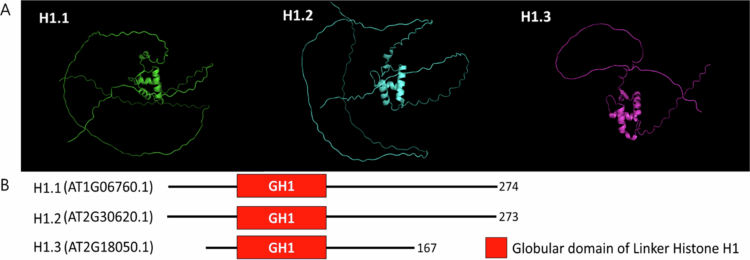
A predicted 3D structures of Arabidopsis H1 variants generated by I-TASSER. (A) Ribbon models of H1.1 (AT1G06760), H1.2 (AT2G30620), and H1.3 (AT2G18050), highlighting the conserved GH1 winged-helix domain (center) and variable *N*-/C-terminal tails. (B) Superposition of GH1 domains (RMSD <  1.5  Å), showing structural conservation. Structures reveal tail flexibility enabling DNA bridging/compaction; confidence scores (C-score > -1.5) indicate high reliability. (Generated from UniProt sequences using I-TASSER v5.1; Zhang Lab). (B) Schematic representation of the domain structures of Arabidopsis histone H1 variants (H1.1, H1.2, and H1.3). All three histone H1 variants contain a centrally located and highly conserved globular domain (GH1, Red box). While H1.1 (274 aa) and H1.2 (273 aa) exhibit typical protein lengths, the stress-inducible variant H1.3 (167 aa) is characterized by significantly shorter *N*-terminal and C-terminal tails. The numbers on the right indicate the total amino acid length of each protein.

Histone H1 (H1), a crucial component of all eukaryotic chromatin, fulfills multifaceted roles in stress responses and epigenetic regulation, as its primary function is chromatin compaction.^14^ ^,^ ^17^ Arabidopsis H1 exhibits diverse characteristics, notably epigenetic and stress adaptation functions.^1^ ^,^ ^2^ H1 comprises a conserved tripartite structure—a short *N*-terminal tail, central GH1 domain, and lysine-rich C-terminal tail—as shown in I-TASSER 3D models (Figure 1A). Such a specific structure enables H1 to bind to nucleosomes at their entry and exit sites, interact with linker DNA, and facilitate chromatin compaction.^2^ ^,^ ^8^ ^,^ ^17^ ^,^ ^18^ Among species, the GH1 is highly conserved, while the tails are varied and contribute to the functional diversity of H1 across eukaryotes (Figure 1B).^8^

**Figure 2.**
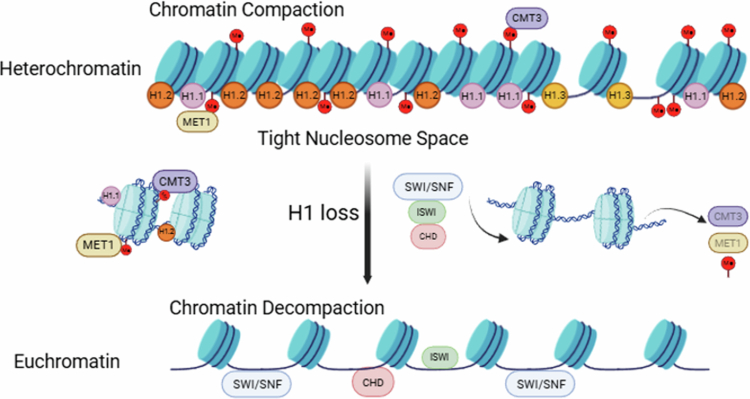
Mechanistic model of H1-mediated chromatin stabilization and epigenetic regulation. Histone H1 stabilizes the nucleosome and promotes higher-order chromatin compaction by binding to linker DNA. The depletion of H1 (h1 mutants) leads to global chromatin decompaction and increased accessibility. This structural shift allows chromatin remodelers (SWI/SNF, ISWI, and CHD) to reposition nucleosomes and facilitates the access of RdDM machinery to previously silenced regions, resulting in altered DNA methylation patterns and the reactivation of transposable elements (TEs). Figure created with BioRender.com.

In *Arabidopsis*, three H1 variants – H1.1(AT1G06760), H1.2(AT2G30620), and H1.3(AT2G18050) – have been identified. H1.1 and H1.2 are identical in canonical forms (Figure 1B), comparable in structure and function, exhibit enrichment in heterochromatin, and are inversely correlated with elevated gene expression levels.^5^ ^,^ ^11^ ^,^ ^17^ In contrast, H1.3 is a stress-inducible variant with shorter *N*- and C-terminal tails and reduced DNA-binding motifs (lacking several STPXK sites), which enhances its mobility along DNA molecules and alters chromatin association dynamics, particularly under drought stress, ABA signaling, and low-light conditions.^19^ ^,^ ^20^ Thus, canonical H1.1/H1.2 act as structural gatekeepers maintaining compact, repressive chromatin under normal conditions, while H1.3 confers stress-induced plasticity for adaptive reprogramming.^5^ ^,^ ^10^ ^,^ ^16^ ^,^ ^17^ ^,^ ^19^ ^,^ ^20^ The H1 functions in chromatin compaction and transcript regulation have been extensively investigated. For instance, H1 promotes the compaction of nucleosome arrays into higher-order fibers to preserve genome stability and suppress transcription when required.^2^ ^,^ ^17^ Additionally, different H1 variants selectively associate with components of chromatin remodeling complexes to enable transcriptional regulation in diverse genomic contexts.^5^ ^,^ ^17^ Post-translational modifications, including phosphorylation and acetylation, are emerging as potential regulators of H1 function, although their comprehensive in vivo characterization in plants remains relatively limited compared to animal systems. Specifically, phosphorylation of H1.1 and H1.2 at serine (Ser2 and Ser13) and threonine residues, alongside acetylation of H1.2 at lysine 17 (K17), has been proposed to modulate their chromatin-binding affinity and gene accessibility.^12^ ^,^ ^18-20^ In contrast, H1.3 phosphorylation is suggested to correlate with chromatin condensation at specific gene loci, such as *RD29A*, during stressful conditions, potentially integrating epigenetic control with stress responses (Figure 2).^5^ ^,^ ^20^ Additionally, H1.3 acetylation facilitates the transcriptional activation of immune-system-related genes such as *PR1* and *WRKY29.* ^19-24^ Recent reports have highlighted the essential roles of H1 in regulating DNA methylation and histone modifications. For instance, H1 occupancy acts as a physical barrier that restricts the genomic access of the DNA demethylase DME (DEMETER). By inhibiting DME-mediated active DNA demethylation, H1 maintains the stability of DNA methylation patterns at specific loci, thereby influencing the transcription of imprinted genes.^2^ ^,^ ^10^ ^,^ ^25-27^ H1 variants can also modulate histone marks, such as H3K27me3, thereby altering gene expression profiles (Figure 2).^15^ ^,^ ^17^ Such a dynamic interplay among H1 variants plays a critical role in the stress-adaptive response of plants. Specifically, the induction of H1.3 allows it to displace the canonical H1 at nucleosomes during drought stress and ABA-based signaling, resulting in chromatin decompaction and elevated accessibility, enabling a rapid activation of stress-responsive genes.^20^ ^,^ ^23^ Conversely, H1.1 and H1.2 maintain genome stability by repressing the negative regulators of gene expression under normal conditions. To merge the dual role of H1, we propose a synthesis based on variant-specific functions and physical chromatin states. Canonical variants H1.1 and H1.2 function as structural repressors by reinforcing heterochromatin stability and DNA methylation. Conversely, the stress-inducible H1.3 facilitates chromatin plasticity under adverse conditions. A critical mechanistic resolution is provided by recent findings that H1 drives heterochromatin condensation through phase separation. This property allows H1 to orchestrate the physical density of heterochromatin, balancing genomic silencing with the structural flexibility required for rapid epigenetic reprogramming during stress.^11^ ^,^ ^20^ ^,^ ^26^ These mechanisms through which H1 variants influence chromatin architecture and gene activity highlight them as potential targets for enhancing crop resilience to climate change and environmental challenges.^2^ ^,^ ^17^ ^,^ ^21^ This review provides a comprehensive overview of the significance of H1-associated epigenetics and stress responses in plants, as well as the recent advances in understanding H1 structure, variant diversity, and their integrated roles in chromatin organization and gene regulation.^17^

### H1 as a chromatin assembly regulator in plants

The dynamic functions of linker histone H1 in associating with and regulating chromatin accessibility are fundamental to maintaining chromatin architecture and gene expression in plants. In *Arabidopsis*, heterochromatic and transposable element (TE)-rich genomic regions are enriched with H1.1 and H1.2. Evidence from Whole-Genome Bisulfite Sequencing (WGBS) and MNase-seq suggests that these variants may promote higher-order chromatin compaction and suppress TE mobility, likely by restricting the access of DNA methyltransferases, such as CMT2, to the DNA within these domains (Figure 2) (Table 1).^5^ ^,^ ^11^ ^,^ ^26^ ^,^ ^28^ Loss-of-function mutations in H1 (*h1* mutants) increase chromatin accessibility, as evidenced by enhanced DNase I hypersensitivity and increased ATAC-seq signals at the promoters of stress-responsive genes, including *WRKY29* and *HAC2*. These observations are consistent with the role of H1 as a structural barrier; its absence enhances innate immunity, elevates ROS production, and activates MAPK-based signaling pathways.^5^ ^,^ ^20^ In addition, current models suggest that H1 inhibits the inclusion of the euchromatic histone variant H2A.Z, which may help preserve the euchromatin–heterochromatin balance by suppressing heterochromatin condensation.^11^ ^,^ ^14^ ^,^ ^16^ ^,^ ^21^ ^,^ ^28^ ^,^ ^29^ Such coordination ensures the maintenance of appropriate chromatin domain borders and accessibility, which are essential for gene silencing and rapid transcriptional activation in response to developmental cues and environmental stimuli.^29^

**Table 1.** Schematic summary of multi-omics features associated with Arabidopsis H1 variants. The table integrates transcriptome, DNA methylome, histone modification, and small-RNA profiles from loss-of-function or induction studies of H1.1, H1.2, and H1.3, highlighting their partially overlapping yet distinct roles in regulating growth, flowering, transposable elements, immunity, and stress-adaptive memory genes. H1.1 and H1.2 primarily associate with TE-rich, CG/CHG-methylated, repressive heterochromatin and 24-nt siRNA-mediated silencing, whereas stress-inducible H1.3 is linked to dynamic DNA methylation, permissive histone marks, siRNA mobilization, and integration of environmental stress signals into chromatin states.

H1 Variant	Localization	Structural feature	Key functions & mechanisms	Post-translational modifications (PTMs)	References
H1.1	Euchromatin & Heterochromatin	Canonical; long CTD	Fine-scale chromatin architecture, developmental timing	Phosphorylation (potential), Ubiquitination (suggested)	[7,12,15,23]
H1.2	Primarily heterochromatin	Canonical; high homology to H1.1	TE silencing, interaction with DDM1 & H2A.W	Acetylation (proposed), SUMOylation (implicated)	[3,11,12,19,30]
H1.3	Stress induced (drought, low light, etc)	Truncated CTD; lacks S/T motifs	Chromatin plasticity, phase separation, redox response	Phosphorylation (ABA-dependent)	[10,15,20,24]
H1 Knockout (h1.1/h1.2/h1.3 triple mutant)	Genome-wide	–	Loss of TE silencing, compensatory H3K9me2	Increased H3K9ac, reduced H3K27me3	[3,4,10,12,28,31]

The PTMs of H1 can also modulate its chromatin regulatory functions; however, compared to animals, their comprehensive characterization in plants remains limited, with evidence primarily emphasizing their roles in stress adaptation.^18^ One such PTM, H1 phosphorylation, particularly of serine residues within the C-terminal domain, is associated with stress-induced chromatin relaxation. Beyond abiotic stress, the post-translational modification (PTM) of H1 variants plays a pivotal role in plant immunity, echoing conserved mechanisms observed in animal systems. In animals, H1 phosphorylation at specific serine/threonine motifs by cyclin-dependent kinases (CDKs) or MAPKs is a hallmark of chromatin remodeling during the cell cycle and stress response. Similarly, a nuclear phosphoproteomic screen identified that Arabidopsis H1.2 is strongly phosphorylated upon flg22 treatment, suggesting it as a potential downstream target of the MAPK signaling cascade. The presence of conserved (S/T)P motifs across both plant and animal H1 variants suggests a universal regulatory code where phosphorylation reduces the positive charge of the H1 C-terminal domain, thereby weakening its affinity for linker DNA and facilitating the rapid transcriptional activation required for biotic defense.^12^

In Arabidopsis, interphase phosphorylation at *N*-terminal Ser/Thr residues (e.g., Ser2 and Ser13) and C-terminal domain sites introduces negative charges, inducing electrostatic repulsion, tail unfolding, and weakened DNA interactions that drive chromatin relaxation for rapid transcriptional responses to drought or ABA signaling.^18-20^ Proteomic analyses identify ~25 distinct PTMs on H1 variants, including conserved acetylation/methylation alongside novel plant-specific crotonylation, propionylation, and formylation on lysine-rich tails, which collectively fine-tune nucleosome spacing, mobility, and phase separation propensity to balance compaction and accessibility.^11^ ^,^ ^17^ ^,^ ^18^ ^,^ ^28^ Notably, the SQ motifs within the C-terminal tail of the heterochromatin-specific H2A.W variant – structurally similar to H1 – are phosphorylated during DNA damage repair, promoting chromatin remodeling within compacted regions.^4^ H1 depletion also affects histone acetylation levels; specifically, reduced H3K56 acetylation triggers immune responses, indicating a potential crosstalk between H1 and histone acetyltransferases or deacetylases, such as HAC2 and HDA18. Thereby priming immunity pathways and illustrating PTM-H1 crosstalk in abiotic/biotic stress integration.^13^ ^,^ ^17^ ^,^ ^18^ ^,^ ^24^ ^,^ ^29-31^ Although direct *in vivo* evidence for H1 acetylation and methylation in plants remains limited, the conservation of crucial structural domains, such as GH1 and the lysine1-rich C-terminus, supports the likelihood that H1 serves as a substrate for various PTMs that regulate its DNA-binding affinity and chromatin structure assembly during stress adaptation (Figure 2).^12^ ^,^ ^17^ ^,^ ^18^ ^,^ ^24^ ^,^ ^30^ Furthermore, the regulatory roles of H1 extend to developmental processes. By stabilizing the chromatin structure through nucleosome binding and chromatosome formation, H1 is proposed to influence DNA methylation and histone modifications.^8^ ^,^ ^11^ ^,^ ^26^ In Arabidopsis, evidence from Whole-Genome Bisulfite Sequencing (WGBS) and Chromatin Immunoprecipitation sequencing (ChIP-seq) is consistent with the model that H1 may facilitate the maintenance of CG and CHG methylation patterns, particularly at heterochromatic regions and TEs.^5^ ^,^ ^9^ ^,^ ^19^ ^,^ ^22^ ^,^ ^23^ ^,^ ^31^ ^,^ ^32^ It also represses H3K4me3 deposition by restricting the access of methyltransferases, such as ATX1, to euchromatic promoters, a regulatory mechanism similar to SET7/9 inhibition in animal systems.^12^ ^,^ ^18^ ^,^ ^28^

H1 depletion has pleiotropic effects, including alterations of stomatal patterns, delayed flowering, increased basal immunity, and enhanced stress-related signaling, which are typified by a hyperactivation of the PTI pathways, elevated ROS contents, and improved MAPK activity.^12^ ^,^ ^19^ ^,^ ^33^ Drought stress activates H1.3, which condenses chromatin at the loci of stress-responsive genes, like RD29A, to modulate ABA-based signaling and redox homeostasis, thereby orchestrating a balance between transcriptional activation and genome stability.^20^ ^,^ ^28^ Notably, H1 also interacts with histone modifiers, such as HDA18 and HAC2, forming a feedback regulatory loop involving chromatin compaction via histone acetylation/deacetylation, which regulates the expression of stress-responsive genes.^19^ Collectively, these diverse actions define H1 as a chromatin “rheostat” that integrates transcriptional reprogramming, epigenetics, and chromatin topological remodeling to fine-tune growth, defense, and stress adaptation in plants.

## Epigenetic functions of H1: DNA methylation and histone modifications

H1 variants influence global and locus-specific DNA methylation: H1 orchestrates DNA methylation patterns in Arabidopsis through context-dependent mechanisms.^10^ The *h1* mutant demonstrates genome-wide CHH hypermethylation at euchromatic loci, including gene families and promoters of stress-responsive genes, such as *WRKY29* and *HAC2,* without compromising heterochromatin stability.^5^ ^,^ ^25^ Additionally, functional cooperativity between H1 and H2A.W is crucial for maintaining heterochromatin structure; both these factors regulate chromatin accessibility and DNA methylation at repetitive sequences.^25^ Loss-of-function mutations in either factor disrupt DNA compaction, increase chromatin accessibility, and impede transposon silencing.^4^ ^,^ ^12^ ^,^ ^25^ ^,^ ^34^ Importantly, loss-of-function H1–H2A.W double mutants display phenotypes suggestive of a pronounced inhibition of TE silencing and genome instability, highlighting their synergistic roles.^4^ ^,^ ^25^ ^,^ ^34^

Compensatory silencing pathways in the absence of H1: H1 mutations disrupt global chromatin compaction, leading to increased accessibility. However, these mutations also trigger compensatory mechanisms to suppress uncontrolled transcription and TE activation.^4^ ^,^ ^5^ ^,^ ^11^ ^,^ ^15^ For instance, despite H1 loss, residual MET1 and CMT3 activities continue to maintain CG/CHG methylation at TEs. Furthermore, increased H3K9me2 deposition, which is mediated by SUVH4/5/6 methyltransferases, has been observed to partially compensate for the loss of silencing.^4^ ^,^ ^5^ ^,^ ^34^ These feedback regulatory mechanisms act as safeguards to maintain genome integrity even in the absence of H1.

Collaborative regulation and localized hypermethylation: H1 and the histone variant H2A.W act cooperatively to maintain heterochromatin structure and regulate DNA methylation at repetitive sequences.^25^ ^,^ ^27^ Loss of both factors results in a pronounced inhibition of TE silencing and significant genome instability. Interestingly, *h1* mutants exhibit a paradoxical genome-wide CHH hypermethylation at specific euchromatic loci, such as the promoters of *WRKY29* and *HAC2*. This phenomenon is driven by unhindered CMT2 or DRM2 activity at regions where H1 previously acted as a physical barrier.

Role of H1 in RdDM and sRNA biogenesis: H1 also modulates the RNA-directed DNA methylation pathway by restricting siRNA biogenesis at euchromatic TE (Table 2). H1 depletion increases DNA accessibility and non-CG methylation, enabling the RdDM machinery to target genes and TEs, which are normally silent.^5^ ^,^ ^9^ Thus, H1 acts as a structural modulator of chromatin plasticity, integrating structural and epigenetic cues to regulate genome stability and transcriptional activity.^7^ ^,^ ^28^

**Table 2.** Functional specialization and regulatory features of Arabidopsis Histone H1 variants. The distinct molecular and biological roles of the three Histone H1 variants (H1.1, H1.2, and H1.3) in *Arabidopsis thaliana*, as well as the pleiotropic effects observed in the triple knockout mutant.

Variant	Transcriptome change (KO/Induction)	DNA methylome/methylation	Histone modifications	sRNA/siRNA dynamics	Networked functions	Stress-induced expression			
						Drought	Low light	Salinity	ABA
H1.1	Growth, TE, flowering, immune genes	TE methylation, CG/CHG-rich	Repressive marks, H3K9me	Suppresses 24nt siRNAs in TEs	Chromatin stability, silencing	Down-regulated	Slightly Down	Down-regulated	No Change
H1.2	Growth, TE, flowering, immune genes	Largely overlapping with H1.1	Repressive marks, H3K9me	Suppresses 24nt siRNAs in TEs	Chromatin stability, silencing	Down-regulated	Slightly Down	Down-regulated	No Change
H1.3	Stress-inducible; enriches ABA/ drought/memory gene programs	Dynamics, context-dependent	Unknown; possibly modulates active marks under stress (hypothesis)	Largely unknown; no direct evidence for H1.3-specific effect on siRNA	Stress adaptation, signaling integration	Up-regulated	Strongly Up	Up-regulated	Up-regulated

### H1 and chromatin remodeling complexes

Role of H1 in nucleosome stability and positioning: H1 stabilizes higher-state chromatin architecture by binding to the nucleosomal linker DNA, promoting chromatin compaction and regulating nucleosome spacing. The *h1* in Arabidopsis allows increased access to chromatin, particularly linker regions, thereby inducing nucleosome repositioning and heterochromatin destabilization.^4^ ^,^ ^5^ ^,^ ^14^ The GH1 anchors H1 to the nucleosome, whereas its lysine-rich C-terminal tails mediate inter-nucleosomal associations that enforce chromatin rigidity (Figure 1B). Genome-wide analyses, such as MNase-seq, have revealed that H1 depletion disrupts nucleosome phasing, reduces heterochromatin stability, and enables ectopic transcription factor binding.^4^ ^,^ ^5^ ^,^ ^14^ ^,^ ^25^

H1 interactions with chromatin remodelers and their effects on transcriptional regulation: H1 serves as a critical structural component and intimately interacts with chromatin-remodeling complexes – including SWI/SNF, ISWI, and CHD – to modulate nucleosome positioning and gene transcription levels. It also contributes to the maintenance of chromatin domain borders by restricting the access of chromatin remodelers, thereby limiting nucleosome sliding and eviction (Figure 2). H1 depletion alters the activity of these chromatin remodelers, resulting in an aberrant chromatin structure and transcriptional misregulation, particularly at TEs and heterochromatic regions (Figure 2).^4^ ^,^ ^5^ ^,^ ^23^

Impact of H1 on Chromatin Dynamics (ATAC- and MNase-seq data): Genome-wide chromatin accessibility assays, such as ATAC-seq, are commonly employed to assess chromatin structure.^4^ ^,^ ^14^ ATAC-seq data demonstrate increased accessibility mainly at heterochromatic regions near TEs, with *h1* exhibiting a reduced chromatin compaction capacity.^4^ Complementary MNase-seq profiling reveals that H1 depletion alters nucleosome positioning, highlighting its pivotal role in the spatial organization of chromatin^4^ ^,^ ^5^ ^,^ ^14^ Collectively, these findings underscore the significance of H1 in maintaining chromatin boundaries and suppressing the aberrant transcriptional activation of TEs.^4^ ^,^ ^5^ More crucially, H1-dependent chromatin structures affect transcriptional reprogramming patterns and the response to environmental cues more rapidly than when they are changed by steady-state and baseline levels of gene expression.^14^

## The role of H1 in plant stress responses

The H1 eviction is a well-documented phenomenon in animal systems, particularly during rapid immune responses, where H1 removal is coordinated by specific chaperones and remodeling complexes to facilitate gene activation. While the specific chaperones for H1 eviction in plants remain to be fully characterized, the observed reduction in H1 occupancy – especially the stress-inducible H1.3 – under abiotic stress suggests that plants may employ a functionally analogous strategy to ensure rapid transcriptional reprogramming.^3^ This highlights a potentially conserved evolutionary theme where H1 stability serves as a primary gatekeeper for environmental and developmental signaling across eukaryotes.^35^ H1 plays a critical role in the response and adaptation of plants to various stresses. We present a point-by-point discussion. Regulation of chromatin accessibility during abiotic stress: Under abiotic stress, H1 plays a dynamic regulatory role in chromatin compaction, which, in turn, helps maintain a balance between transcriptional activation and genome stability.^16^ ^,^ ^26^ ^,^ ^28^ In drought conditions, H1.3 (a stress-inducible H1 variant) promotes the condensation of chromatin at specific loci, such as *RD29A*, thereby enhancing ABA-dependent signaling and ROS homeostasis while maintaining photosynthetic efficiency.^7^ ^,^ ^20^ ^,^ ^33^ Under cold stress, H1 depletion increased nucleosome eviction at COR15A promoters, thereby enabling rapid transcriptional activation without the excision of repressive histone marks such as H3K27me3.^15^ ^,^ ^19^ ^,^ ^36^ Furthermore, H1 interacts with H2A.W to regulate heterochromatin stability under salt stress, a phenomenon that efficiently prevents transposon reactivation to maintain non-CG methylation.^4^ ^,^ ^23^ ^,^ ^25^ ^,^ ^34^ Such an action of H1 ensures the maintenance of genomic integrity under specific conditions.^4^ ^,^ ^5^ These molecular mechanisms highlight H1 function as a chromatin organizer that modulates the accessibility of stress-responsive genes and preserves genome integrity.^4^ ^,^ ^7^ ^,^ ^28^

### H1 variants have distinct roles in stress adaptation

H1 variants exhibit functional specialization: H1.1 or H1.2 are responsible for maintaining heterochromatin silencing at TEs via MET1/CMT3-mediated CG/CHG methylation.^3^ ^,^ ^5^ ^,^ ^26^ ^,^ ^32^ In contrast, H1.3 is induced by drought, ABA, and low light to fine-tune stomatal movements and redox balance.^20^ ^,^ ^28^ The triple mutation *3h1* promotes basal levels of innate immunity and restores impaired stress memory, indicative of H1.3’s unique role in immune priming.^19^ ^,^ ^26^ H1.1/H1.2 depletion increases euchromatin accessibility, hyperactivating *PR1* and *RBOHD* during pathogen infection.^19^ In contrast, H1.3 competes with canonical H1 to alter chromatin dynamics under osmotic stress.^26^ ^,^ ^28^ Such functional divergence between H1 variants enables plants to express the unique responses to specific stressors. The functional divergence among H1 variants, particularly between the highly homologous H1.1 and H1.2, is primarily driven by their distinct biochemical properties and post-translational modification (PTM) landscapes. While H1.1 and H1.2 share over 90% amino acid identity, their chromatin residence time and binding affinity differ owing to subtle variations in their intrinsically disordered C-terminal domains (CTD). Unlike canonical H1s, the stress-inducible H1.3 possesses a truncated CTD and lacks several key DNA-binding motifs, which results in a significantly lower binding affinity and facilitates a more dynamic, “open” chromatin state during environmental stress.^18^

The dynamic regulation of H1 variants is not only crucial for immediate stress adaptation but also appears to play a significant role in stress memory. H1 occupancy and its associated epigenetic marks are instrumental in maintaining a molecular “memory” of previous environmental challenges. Specifically, remodeling of H1-mediated chromatin states during an initial stress encounter can facilitate a faster and more robust transcriptional response upon re-exposure. This strategy, governed by H1-dependent chromatin accessibility, suggests that H1 acts as a central coordinator for transgenerational stress memory, ensuring plant resilience in fluctuating environments.^13^ ^,^ ^24^

### Epigenetic reprogramming via H1 depletion and histone acetylation/methylation changes

The loss-of-function in the H1 allele triggers genome-wide CHH hypermethylation at euchromatic loci (e.g., *WRKY29* promoters) through unhindered CMT2/DRM2 activity.^5^ ^,^ ^28^ During flg22 infection, the *3h1* mutation suppresses H3K56ac levels and enhances DNA methylation, thereby repressing transcriptional responses.^19^ In addition, H1 depletion altered interactions with histone modifiers. For instance, HAC2 (acetyltransferase) and HDA18 (deacetylase) exhibit dysregulated expression, which hinders the acetylation and deacetylation cycles necessary for stress memory.^19^ ^,^ ^28^ ^,^ ^33^ These alterations suggest that H1 protects epigenetic plasticity and ensures dynamic gene regulation under recurrent stresses by restricting DNA methyltransferase access and maintaining histone acetylation levels.^4^ ^,^ ^16^ ^,^ ^19^ ^,^ ^28^ ^,^ ^33^ Furthermore, these variants are subject to differential PTMs that function as a molecular code; for instance, H1.1 is predominantly modified by specific acetylation sites that regulate its turnover, whereas H1.2 undergoes robust phosphorylation in response to biotic signals, such as flg22-triggered MAPK pathways. These site-specific modifications alter the electrostatic interaction between the H1 CTD and the linker DNA, providing a mechanistic basis for how H1.3 can displace canonical variants or how homologous H1s can orchestrate non-redundant epigenetic programs.^18^

### H1 mutation leads to transcriptional activation of stress-responsive genes

The diverse roles of H1 variants (H1.1, H1.2, and H1.3) in plant stress responses have become increasingly apparent. Each variant exhibits distinct expression patterns and functional properties in response to various stresses. For instance, H1.3 levels elevate and promote ABA-mediated responses under drought conditions.^20^ Furthermore, PTMs of H1 (phosphorylation, acetylation, and methylation) are involved in the fine-tuning of stress responses. PTMs also alter the binding affinity of H1 to chromatin as well as other regulatory proteins, facilitating the recruitment of chromatin-remodeling complexes or modulating the accessibility of the transcriptional machinery to target promoters, thereby altering the expression of stress-responsive genes.^3^ ^,^ ^8^ ^,^ ^17^

Loss-of-function in H1 constitutively activates stress-responsive genes (*RBOHD*, *PR1*) via chromatin loosening, thereby promoting basal levels of ROS and MAPK-based signaling.^19^ ^,^ ^31^ ^,^ ^33^ However, in the *3h1* mutant, flg22 treatment was unable to stimulate *WRKY29* and *MAPKKK15* owing to DNA hypermethylation and stagnant H3K56ac levels at their promoters.^19^ This contradiction emphasizes the dual roles of H1; it suppresses baseline transcription but facilitates stress-induced chromatin remodeling.^5^ ^,^ ^19^ The decline in HDA18/HAC2 expression seen in the *3h1* mutant adds another dimension to H1 functions: maintaining a balance between stress signaling and epigenetic regulation. Such an approach highlights constitutive defense and primed responsiveness.^19^ ^,^ ^28^

### H1 as an epigenetic barrier to RdDM machinery access

Beyond its structural role, H1 serves as a critical epigenetic gatekeeper by defining the boundaries of DNA methylation.^5^ ^,^ ^10^ ^,^ ^11^ Recent landmark studies have revealed that H1 acts as a physical barrier restricting the access of the RNA-directed DNA methylation machinery to specific loci.^5^ ^,^ ^9^ In *h1* mutants, increased chromatin accessibility, especially at linker DNA regions, facilitates the ectopic recruitment of RNA Polymerase IV (Pol IV) and subsequent recruitment of the RdDM apparatus.^5^ ^,^ ^14^ This mechanism leads to a paradoxical phenomenon where certain euchromatic regions and TE boundaries undergo localized CHH hypermethylation, despite an overall trend of heterochromatin relaxation (Table 2).^4^ ^,^ ^5^ Furthermore, H1-mediated compaction is essential for preventing any aberrant Chromomethylase 2 (CMT2) activity, which would otherwise gain interactability with previously inaccessible regions within the genomes of *h1* mutants.^5^ ^,^ ^10^ ^,^ ^32^ This sophisticated regulation mechanism ensures that stress-responsive genes maintain a precise epigenetic state; without H1, the resulting “epigenetic noise,” characterized by off-target DNA methylation, can impair the plant’s ability to mount a coordinated transcriptional response to environmental stimuli.^5^ ^,^ ^26^ Consequently, H1 not only merely maintains gene silencing but orchestrates a complex “epigenetic controller” that balances chromatin openness with genomic integrity.^4^ ^,^ ^28^

### H1 variants and a multi-omics perspective in Arabidopsis

Multi-omics technologies have recently enabled a thorough analysis of the functional specialization of H1 variants in Arabidopsis. As mentioned earlier, H1 has three major variants – H1.1, H1.2, and H1.3 – and each one exhibits unique patterns of expression, chromatin association, and regulatory function. Multi-omics studies integrating transcriptomics, epigenomics, proteomics, and chromatin accessibility analysis shed light on the nuanced specializations of these H1 variants throughout developmental processes and stress responses, providing comprehensive and system-level perspectives (Table 1).^14^ ^,^ ^23^ ^,^ ^26^ ^,^ ^28^

Structurally, the three H1 variants share the GH1 domain, which is responsible for nucleosome binding; however, their distinct N- and C-terminal regions promote diversity in regulatory dynamics.^28^ H1.1 and H1.2 are constitutively expressed and tend to associate with heterochromatin, whereas H1.3 is stress-inducible, usually expressed under drought stress, ABA accumulation, and low light. These unique features of H1.3, such as stress-specific induction and distinct sequence motifs, enable it to modulate chromatin accessibility for fine-tuning various processes, including stomatal movements and redox balance (Table 1).^20^ ^,^ ^31^ ^,^ ^37^

At the transcriptome level, loss-of-function in H1.1 and H1.2 (double mutants) or in all three variants (triple mutants, "*3h1*") alters gene expression patterns, such as the upregulation of defense- and hormone-based signaling-related genes, even under non-stress conditions.^3^ ^,^ ^19^ ^,^ ^28^ Notably, H1.3 induction can trigger the activation of stress-responsive genes – such as *RD29A* – acting in synergy with classic abiotic stress-responsive pathways like ABA-based signaling.^3^ ^,^ ^20^ ^,^ ^28^

From an epigenomic perspective, H1.1 and H1.2 play crucial roles in maintaining repressive DNA methylation levels at TEs via MET1 and CMT3 activity, thereby preserving heterochromatin compaction and genome stability (Table 1).^5^ ^,^ ^26^ In euchromatic regions, H1 depletion leads to extensive abnormalities in DNA methylation patterns, which often promote hypermethylation at TEs and substantial alterations in the expression of genes linked with defense and development.^5^ ^,^ ^19^ The H1.3 variant dynamically moves to more accessible chromatin domains under stress-inducing conditions, supporting context-dependent DNA methylation alterations that facilitate transcriptional reprogramming.^26^ ^,^ ^28^

Loss of H1 function, particularly in H1.1/H1.2, also influences the small-RNA landscape in plants by alleviating the repression of non-CG methylation-coupled 24-nt siRNA production in heterochromatin, a process mainly constrained by H1 (Table 2).^5^ This observation highlights the role of H1 as a boundary element controlling the interface between RdDM and chromatin domain stability. Chromatin accessibility assays, such as ATAC-seq, have further elucidated the impacts of H1 variants. While loss of H1.3 function elevates inter-nucleosome spacing and the exposure of formerly silenced regions, mutations in H1.1 and H1.2 restrict access at heterochromatin and TE borders. In triple mutants, these structural changes are mirrored by functional consequences, which increase chromatin openness at the loci of key immune-system-related and stress-responsive genes to exhibit hypersensitivity or priming defects in stress responses.^4^ ^,^ ^5^ ^,^ ^19^ ^,^ ^28^ Protein interaction network analyses suggest that H1.1 and H1.2 mainly associate with canonical chromatin remodelers (e.g., DDM1 and CAF-1) and DNA methyltransferases (MET1 and CMT3) to enforce gene silencing and maintain chromatin stability (Table 1).^4^ ^,^ ^7^ ^,^ ^25^ In contrast, H1.3 interacts with proteins involved in transcriptional activation, stress signaling, and hormonal pathways, facilitating the environmental challenge response in plants.^19^ ^,^ ^28^ Each variant contributes to a distinct set of associations that influence the overall chromatin architecture and functional transitions.^20^ ^,^ ^28^

These observations reveal the processes by which Arabidopsis H1 variants operate as the conservators or modulators of the chromatin state. H1.1 and H1.2 safeguard developmental stability through repression, while H1.3 endows the plant with remarkable epigenetic plasticity and stress adaptability.^38^ Such an intricate division of labor among H1 isoforms deciphered through multi-omic integration underscores their fundamental importance in the context of growth, defense, and epigenome dynamics in plants (Table 1).^4^ ^,^ ^5^ ^,^ ^7^ ^,^ ^12^ ^,^ ^16^ ^,^ ^19^ ^,^ ^20^ ^,^ ^28^

Multi-omics analyses facilitate the precise attribution of specialized and complementary functions to each H1 variant, thereby illuminating both the constitutive and stress-responsive aspects of chromatin architecture in Arabidopsis.^19^ ^,^ ^31^ This approach dynamically interplays canonical H1 variants H1.1 and H1.2 to enforce epigenetic silencing and preserve genome integrity. However, the stress-inducible H1.3 variant confers chromatin plasticity and adaptability to adverse environmental challenges.^5^ ^,^ ^11^ ^,^ ^16^ ^,^ ^20^ ^,^ ^35^ Since Arabidopsis encodes environmental memory and developmental regulation at the chromatin level, H1 variants serve as molecular rheostats coordinating the balance between transcriptional repression and induced reprogramming.^19^ ^,^ ^20^

The interplay between H1 and other chromatin marks, such as the histone variant H2A.Z and histone H3K27me3, is established as a pivotal modulator of dynamic and stress-specific chromatin states. Loss of H1 function alters the genome-wide distribution of H2A.Z, particularly at TEs and the promoters of stress-responsive genes, influencing their silencing/activation patterns.^14-16^ ^,^ ^21^ H1 depletion promotes the redistribution of H3K27me3, which is deposited by polycomb repressive complexes. This entire mechanism influences the expression of development-associated and stress-responsive genes by repressing the histone marks at their loci, such as COR15A.^14^ ^,^ ^15^ ^,^ ^21^ ^,^ ^39^ The study signifies the crosstalk between H1, H2A.Z, and H3K27me3 as a mechanism involved in orchestrating maintenance of chromatin boundaries and flexible stress-inducible gene expression, which supports the notion of context-dependent chromatin states in plants (Table 2).^10^ ^,^ ^15^ ^,^ ^16^ ^,^ ^21^ ^,^ ^28^ ^,^ ^29^

### Future perspectives

Despite considerable progress, a comprehensive integration of H1 modifications within the broader epigenetic landscape in plants remains an intriguing area of study. The linker protein H1 serves as a central orchestrator bridging DNA methylation, diverse histone modifications, and global chromatin accessibility to uphold genome integrity and transcriptional flexibility under stress-inducing conditions.^7^ ^,^ ^31^ H1 physically interacts with DNA methyltransferases such as MET1 and CMT3, ensuring TE silencing while simultaneously constraining excessive methylation of euchromatic regions.^5^ ^,^ ^19^ PTMs – phosphorylation and acetylation – further explain the recruitment of acetyltransferases (e.g., HAC2) or deacetylases (e.g., HDA18) by H1, which modulates gene expression in response to stress.^20^ ^,^ ^28^ ^,^ ^31^ However, the precise mechanisms by which H1 PTMs intersect with histone marks such as H3K4me3 and H3K27me3, or influence the dynamic redistribution of variants such as H2A.Z, remain unresolved.^7^ ^,^ ^16^ ^,^ ^27^ ^,^ ^28^ ^,^ ^36^ ^,^ ^38^ In animals, H1 methylation (e.g., H1.4K26 by G9a) has been linked to HP1-dependent Polycomb silencing; however, comparable mechanisms in plants are still elusive.^8^ ^,^ ^11^

Investigations are underway into the role of H1 in crop species such as rice and wheat. In *Oryza sativa*, the suppression or modification of OsH1.2 alters TE methylation patterns, promotes chromatin relaxation, and results in developmental abnormalities closely resembling those observed in *Arabidopsis*. In wheat (*Triticum aestivum*), the stress-inducible H1 variant is associated with an increased tolerance to drought and salinity and is associated with conserved PTM patterns and chromatin remodeling at the loci of stress-responsive genes.^17^ ^,^ ^20^ ^,^ ^36^ These insights serve to use H1 biology for crop improvement, such as variant-specific PTMs, dynamic chromatin states, and targeted genome editing to improve resilience in agricultural species.^17^ ^,^ ^36^ Specifically, targeted genome editing using CRISPR/Cas9 systems offers a promising avenue to manipulate H1 variants, such as enhancing the expression of stress-inducible H1.3 or precisely modifying its PTM-associated residues to foster a more flexible chromatin state under adverse conditions. However, such translational efforts must navigate significant challenges, including potential pleiotropic effects on plant growth and biomass, as H1 is deeply involved in developmental transitions. Furthermore, global H1 manipulation could trigger epigenetic instability or unintended transposable element reactivation, requiring tissue-specific or stress-inducible promoter systems to ensure that enhanced resilience does not come at the cost of yield or genomic integrity.

Long-term consequences of loss of H1 functions in plants, such as Arabidopsis, include prominent chromatin disruption, including genome-wide DNA hypermethylation, global heterochromatin relaxation, and a partial derepression of TEs.^4^ ^,^ ^5^ These changes provoke epigenetic instability, as constitutively methylated promoters of defense-related genes constrain transcriptional plasticity and undermine an effective response to recurrent stress.^16^ ^,^ ^24^ The *3h1* mutants display altered stomatal movements and root system development patterns, reflecting a fundamental shift in the growth-defense balance.^19^ ^,^ ^27^

Using H1 variants to develop stress-tolerant plants: H1.3 emerges as a dynamic regulator under drought and with ABA exposure, modulating ROS balance and chromatin condensation at loci of stress-responsive genes such as *RD29A,* ^20^ ^,^ ^33^ ^,^ ^36^ while H1.1 and H1.2 serve as steadfast custodians of heterochromatin and establish a threshold for activating defense-related genes.^5^ ^,^ ^24^ CRISPR technology may enable the precise modification of H1.3 to achieve stress-inducible regulation that fine-tunes chromatin elasticity.^20^ ^,^ ^36^

While H1's role in linear occupancy is well-documented, its impact on the three-dimensional (3D) nucleome in plants remains an open frontier. Drawing from insights in animal models where Hi-C analysis revealed H1 as a master regulator of Topologically Associating Domains (TADs) and higher-order folding,^11^ it is imperative to apply similar 3D genomics approaches to plants. Future research utilizing Hi-C will determine whether stress-induced H1 dynamics trigger large-scale reorganization of chromatin domains to facilitate rapid transcriptional reprogramming.^11^

While the integration of high-throughput sequencing technologies has revolutionized our understanding of H1, it is essential to consider the inherent limitations of these experimental approaches. For instance, MNase-seq and ATAC-seq, widely used to assess chromatin accessibility, can be subject to sequence-specific digestion biases and varying sensitivities in heterochromatic regions, which may under-represent H1-stabilized domains.^40^ Similarly, although bisulfite sequencing provides a global map of DNA methylation, it cannot readily distinguish between 5-methylcytosine (5mC) and 5-hydroxymethylcytosine (5hmC), potentially masking nuanced epigenetic layers influenced by H1. Acknowledging these technical constraints is crucial; therefore, a multi-omic approach that combines high-resolution mapping with biophysical assays will be necessary to definitively characterize H1’s multifaceted roles in the plant.^30^ ^,^ ^36^

Future research must disentangle variant-specific and PTM-dependent regulatory roles of H1 under authentic environmental contexts by integrating high-resolution mapping techniques to delineate chromatin-state dynamics.^7^ ^,^ ^28^ Investigating the crosstalk between H1 and alternative histone variants like H2A.Z is critical to understanding how plants orchestrate the modulations in genome architecture.^19^ ^,^ ^27^ ^,^ ^36^
